# Exploring pathways to outpatients’ satisfaction with health care in Chinese public hospitals in urban and rural areas using patient-reported experiences

**DOI:** 10.1186/s12939-019-0932-3

**Published:** 2019-02-06

**Authors:** Xuanxuan Wang, Jiaying Chen, Bo Burström, Kristina Burström

**Affiliations:** 1 School of Health Policy and Management, Nanjing Medical University, Nanjing, China; 2 Health Outcomes and Economic Evaluation Research Group, Stockholm Centre for Healthcare Ethics, Department of Learning, Informatics, Management and Ethics, Karolinska Institutet, Stockholm, Sweden; 3 Centre for Health Policy Studies, Nanjing Medical University, Nanjing, China; 4 Creative Health Policy Research Group, Nanjing Medical University, Nanjing, China; 5 Equity and Health Policy Research Group, Department of Public Health Sciences, Karolinska Institutet, Stockholm, Sweden

**Keywords:** Patient satisfaction, Patient-reported experiences, Public hospitals, China, Structural equation modeling

## Abstract

**Background:**

This study aimed to measure outpatients’ general satisfaction with and experiences of different aspects of health care in Chinese public hospitals and to investigate to what extent general satisfaction could be explained by patients’ experiences in public hospitals located at urban and rural areas.

**Methods:**

Data on 4782 outpatients were derived from a patient survey in 9 city-level (urban) and 16 county-level (rural) public hospitals across China in 2016. According to Donabedian’s model, questions on patients’ experiences were categorized into six aspects under “structure” and “process”, with general satisfaction representing “outcome”. The Chi-square tests were used to test the differences in patients’ experiences and general satisfaction between urban and rural areas. The Partial Least Squares Structural Equation Modeling (PLS-SEM) was used to estimate effects of patients’ experiences on general satisfaction.

**Results:**

Compared with respondents in rural areas, there were significantly higher percentages of respondents in urban areas reporting satisfaction and positive experiences in most aspects. As manifested by the path coefficients in PLS models, the positive effect of professional competence (0.197) on general satisfaction was the most significant in respondents at urban areas, followed by communication and information (0.183), and caring attitudes and emotional support (0.174). Among respondents at rural areas, the positive effect of environment facilities (0.199) was the most significant, followed by caring attitudes and emotional support (0.188), and professional competence (0.179). The PLS models explained 44.9 and 46.0% of variations in patient satisfaction at urban and rural areas, respectively.

**Conclusions:**

Levels of patient satisfaction and experiences at Chinese public hospitals were higher in urban than in rural areas. Outpatients’ experiences of professional competence, caring attitudes and emotional support were strongly related to their satisfaction in both settings. However, among respondents in urban areas, experiences of communication and information were more strongly related to satisfaction, whereas among respondents in rural areas, experiences of environment and facilities were more strongly related to satisfaction.

**Electronic supplementary material:**

The online version of this article (10.1186/s12939-019-0932-3) contains supplementary material, which is available to authorized users.

## Background

Patient satisfaction with health care is an important and commonly used indicator of quality [1]. Research has demonstrated that satisfied patients are more willing to comply with doctors’ instructions, thereby improving positive health outcomes [2–4]. In the literature it is also suggested that satisfied patients are more likely to continue utilizing health services, maintain the relationship with the same provider, and recommend health services to others [5].

Despite its importance, patient satisfaction has been regarded as difficult to interpret, as satisfaction is a multidimensional concept. To improve the quality of care, it would be more fruitful to look at the underlying components [6]. Patient-Reported Experience Measures (PREMs) can capture the underlying components, i.e. patients’ perception of their personal experiences of various aspects of the health care they have received [7], which can help identify tangible priorities and offer actionable indicators for quality improvement [8, 9]. Therefore, measuring and relating the general satisfaction to patients’ experiences can lead to an in-depth understanding of patient satisfaction and highlight the opportunities to improve health care [10].

The Chinese central government initiated a nationwide health care reform in 2009, with the pilot public hospital reform as one of its core reforming areas [11]. Unlike international practice, Chinese public hospitals, which were usually large secondary or tertiary hospitals, provided 92% of outpatient visits across the country and there were no formal referral systems [12, 13]. Patients in China would go to public hospitals even for minor conditions and they generally had low trust in the quality of health care provided in primary health care facilities [13, 14]. The aim of the pilot reform was to improve quality and efficiency of health care services and to control growth of health expenditures in public hospitals [14]. Despite substantial investment to primary health care facilities and the reform on separating government’s operational control and regulatory oversight of public hospitals in China, large secondary and tertiary public hospitals continue to dominate China’s health care delivery [15, 16]. Besides, the allocation of health care resources differ between urban and rural areas in China [17]. In terms of financial resources, from 1995 to 2013, the total health expenditure in China’s urban areas has increased from 123.95 billion yuan to 2.36 trillion yuan, while the corresponding figures in rural areas are 91.56 billion yuan in 1995 and 0.80 trillion yuan in 2013 [18]. The distribution of health human resources also varies tremendously between urban and rural areas. In 2014, the number of health professionals, licensed doctors and registered nurses per 1000 population were 9.70, 3.54 and 4.30 in urban areas respectively, and only 3.77, 1.51 and 1.31 in rural areas respectively [18]. There is also a wide gap in material resources. In 2014, the number of beds in the health care facilities was 2.90 and 2.05 million in the urban and rural area respectively [18]. In terms of evaluation on the public hospital reform in China, clinical quality, technical efficiency, and health expenditures have already been reported in the literature [19–21], but studies on how patients perceive the health care they receive in Chinese public hospitals remain rare, not to mention the disparity in patients’ experiences between urban and rural areas [22]. Increased knowledge in this area from a patient perspective is needed in order to better inform and further guide the public hospital reform.

## Conceptual framework

This study aimed to: 1) measure outpatients’ general satisfaction with and experiences of different aspects of health care provided in Chinese public hospitals; and 2) investigate to what extent general satisfaction with care is explained by patients’ experiences of specific aspects of care in public hospitals in China’s urban and rural areas.

**Fig. 1:**
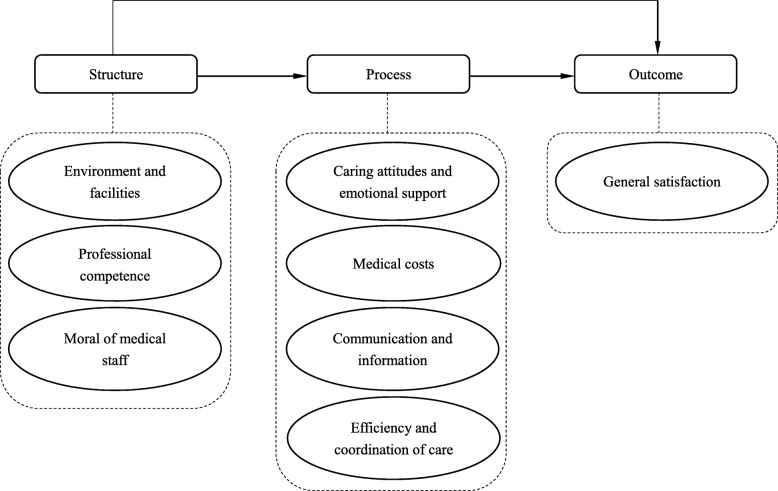
The conceptual model adapted from Donabedian’s model

To fulfill the study aims presented above, an adapted version of Donabedian’s model of quality of care was applied to the present study (Fig. 1). Donabedian’s model was used in a previous qualitative study of our research group to categorize patients’ principal concerns of care provided in Chinese public hospitals [23]. According to the framework and results in the previous study, patients’ experiences of care in public hospitals can be divided into three categories: “structure,” “process,” and “outcome [24].” As depicted in Fig. 1, under the “structure” category, *environment and facilities*, *professional competence* and *moral of medical staff* were identified to be the aspects that patients cared about most in Chinese public hospitals. Under the “process” category, *caring attitudes and emotional support*, *medical costs*, *communication and information*, and *efficiency and coordination of care* were found to be the aspects that patients were most concerned with. Since no questions pertaining to the health outcomes of outpatients were included in the questionnaire, the “outcome” category was adapted into “general satisfaction”, which referred to patients’ general satisfaction with the services provided in public hospitals.

As in Donabedian’s framework, “structure” aspects have direct influence on “process”, and “process” aspects have direct influence on “outcome” [25]. Based on such associations, we hypothesized that in terms of patients’ experiences of care, “structure” and “process” aspects may have direct effects on the general satisfaction, while “structure” aspects may also have indirect effects via “process” on the general satisfaction. This hypothesized relationship is presented in Fig. 1.

## Methods

### Setting

Data in this cross-sectional study were derived from a patient survey conducted in 25 Chinese public hospitals in July and August 2016. Consultations with officials from China’s National Health and Family Planning Commission (NHFPC) and stratified sampling were used to select sampled public hospitals.

In the first step, the research group consulted with officials from the System Reform Division of NHFPC on which cities (in urban areas) and counties (in rural areas) that had implemented tangible reform policies in their public hospitals. As recommended by the NHFPC officials, five cities were selected for the present study, i.e. Zhenjiang in Jiangsu province, Wuhu and Ma’anshan in Anhui province, Baoji in Shaanxi province, and Sanming in Fujian province. In rural areas, two counties from Zhenjiang, Wuhu, Baoji and Sanming were selected respectively.

In the second step, another four cities from the same provinces were selected according to the economic development and population size, i.e. Yangzhou in Jiangsu, Anqing in Anhui, Hanzhong in Shaanxi, and Longyan in Fujian. These cities had not initiated the public hospital reform before the present study was conducted. In rural areas, two counties from each city were selected based on the same criteria. None of the eight counties had implemented the public hospital reform.

In the third step, the biggest municipal public hospital in each sampled city and the biggest county general hospital in each sampled county were selected as sampled hospitals. Altogether, 9 city-level public hospitals and 16 county-level public hospitals were sampled in this study.

### Participants

It was planned to select a convenience sample of 200 outpatients in each sampled hospital to conduct face-to-face interviews with a questionnaire designed by the research group. The inclusion criteria of participants were that outpatients should have completed their visits. The exclusion criteria were that participants could not answer themselves. The original number of outpatients participating in the survey was 4835. After data cleaning, a total of 4782 outpatients were included in the analysis.

### The survey instrument

The questionnaire used in the survey consists of two parts. The first part contains 12 questions on sex, age, socioeconomic status, health insurance, type of disease, and self-rated seriousness of the disease. The second part comprises 43 questions on patients’ experiences of care provided in Chinese public hospitals.

To design the 43 questions concerning patients’ experiences in public hospitals, a qualitative study using patient interviews with open-ended questions was firstly conducted to identify what patients cared most about in China’s public hospitals [23]. Secondly, a pool of relevant and validated questions regarding patients’ experiences of hospital care were identified through an extensive literature review [26–31]. Each question and its corresponding answering options were then translated and adapted to the Chinese language and context. Thirdly, the pool of questions were compared to the findings in the previous qualitative study [23] to ensure that the most frequently mentioned aspects of care by patients themselves were included. In the final step, the original version of the questionnaire was designed and tested among a group of inpatients in one of the city-level public hospitals in Nanjing, the capital city of Jiangsu Province. Minor changes were made according to the feedback from the pilot study to facilitate better understanding of the questionnaire. The final 43 questions included in the questionnaire and their corresponding answering options formulated are shown in Additional file 1: Appendix Table A1.

### Data collection and quality control

A group of five trained interviewers were responsible for all interviews in one sampled city, mainly including patient interviews in one city-level hospital and two county-level hospitals. In each group, a teacher from Nanjing Medical University (NMU) or a member from the research group was appointed the group leader, and group members were master students from NMU. Before the survey, a training workshop was held with all interviewers. Goals and objectives of the survey, inclusion criteria of patients, the minimum requirement of sample size, the survey instrument, and interpersonal interview techniques were explained in the workshop. Pilot interviews were performed within each group, and the group leader was responsible for ensuring consistency in understanding the survey instrument and proficiency in applying the interview techniques.

Face-to-face individual interviews were performed in sampled hospitals. Each interview lasted for 20–30 min. At the end of each day, group members worked in pairs to check the completeness and the logic correctness of each questionnaire. The group leader then double-checked all questionnaires. If information was missing for inpatients, the responsible interviewer went back to the hospital at the same day or the next day to ask the question again.

### Ethical consideration

As only individual interviews were performed without doing any research on human bodies through modern physical, chemical or biological approach, it was unnecessary to seek ethics approval for this study according to *The Ethical Review Policy of Human Biomedical Research* issued by China’s former Ministry of Health [32]. Although no official ethics approval was sought for this study, standard procedures were followed to obtain oral informed consent from interviewees. Having found a potential interviewee according to the sampling criteria, the interviewer introduced his/her own identity and the purpose of the interview. The interviewer then explained that this study was funded by the National Natural Science Foundation of China, which means that the hospital where the interview would be conducted had no influence on the study. The potential interviewee was also informed that his/her participation was voluntary and anonymous and no private information would be asked during the interview. The interviewers also informed that the potential interviewee had the right to refuse to participate in the interview and the right to terminate participation at any time during the interview. If the potential interviewee authorized the interview, to avoid courtesy or gratitude bias, the interview would be conducted when the potential interviewee had completed his/her clinic visit without another third person on spot. Oral informed consent was obtained from each participant.

### Data analysis

#### Measurements of patients’ experiences of care

In the survey, the 43 questions were neither sorted in the order of Donabedian’s model nor categorized by the format of answering options. For data analysis, the 43 questions were firstly reordered based on Donabedian’s model and the aspects depicted in Fig. 1. Next, questions under each aspect were categorized according to their formats of answering options (Additional file 1: Appendix Table A2). Finally, 15 questions of more importance to outpatients themselves were included in the analysis by comparing each question with the findings in the previous qualitative study [23]. The inclusion and exclusion procedures were illustrated in Additional file 1: Appendix Table A3.

The 15 questions included in data analysis are shown in Table 1. A 1–5 Likert style of answering options was applied to all questions, with 1 representing the most positive answer and 5 representing the most negative answer.

**Table 1:** Aspects and questions included in the survey measuring patients’ experiences of care in Chinese public hospitals

Aspects	Questions
Environment and Facilities	Q25. Do you think the hospital environment is comfortable and clean?
	Q27. Do you think the orders in the hospitals are maintained well?
	Q31. Is the layout of different departments reasonable so that outpatients can travel less in the hospital?
	Q36. Could the facilities in the hospital satisfy your need?
Professional competence	Q40. What do you think of the skills of the medical professionals in this hospital?
Caring attitudes and emotional support	Q18. Were the medical professionals friendly and respectful during this visit?
	Q19. Did the doctor listen to the description of your condition patiently during this visit?
Medical costs	Q46. Do you think the amount of money you spent this time was worthwhile regarding to your condition?
	Q47. Do you think the amount of money you spent on registration, diagnosis and treatment this time was reasonable?
Communication and information	Q13. Did the doctor explain your condition and related issues concretely?
	Q21. Did the medical professionals inform you of matters that need attention during the treatment?
	Q22. Are you satisfied with the communication between you and the medical professionals during this visit?
Waiting time	Q42. Do you think the waiting time for registration and paying fees was reasonable?
	Q43. Do you think the waiting time for seeing the doctor was reasonable?
General satisfaction	Q53. In general are you satisfied with the services in this hospital?

### Statistical analysis

The multivariate regression analyses were used to estimate how general satisfaction varied with sex, age, education level, income group, and hospital location. Dummy variables were created for age group, education level, and income group. The Likert-style answering options of all 15 question were converted into dichotomized variables for data analyses, with the most positive and positive answers combined as “positive responses” and the rest as “negative responses”. The χ^2^ tests were then used to test the differences in respondents’ experiences of and general satisfaction with health care provided in public hospitals between urban and rural areas.

Structural Equation Modeling (SEM) was applied in this study, as patients’ experiences cover multiple aspects, and SEM is capable of simultaneously modeling multiple independent variables and multiple dependent variables, estimating the latent variables that the observed variables are meant to measure, and capturing the chains of both direct effects and indirect effects [33].

Among different SEM methods, partial least squares (PLS) was applied in this study. PLS is a component-based SEM technique well suited to assessing complex predictive models [34]. Unlike the covariance-based SEM methods, which require a multivariate normal distribution of the observed variables, PLS is based on the resampling procedures of bootstrapping, which does not make parametric assumptions [35]. Thus, PLS was selected for this present study. PLS models are formally defined by two sets of equations: the measurement model and the structural model. The measurement model specified the relations between a latent variable and its observed variables, whereas the structural model specified the relationships between the latent variables [35].

Several model fit indices should be assessed before interpreting the PLS modeling results. Firstly, to determine the approximate model fit, the standardized root mean square residual (SRMR) should be evaluated. SRMR is the square root of the sum of the squared differences between the model-implied and the empirical correlation matrix [36]. A SRMR value under 0.08 indicates an adequate fit [36].

Secondly, the construct reliability should be assessed. The most important and the only consistent reliability measure for PLS is *ρ*_*A*_ [37]. The other commonly used reliability measures are Cronbach’s *α* and the composite reliability [38]. A minimum value of 0.70 is recommended for all the three measures [35].

Thirdly, the convergent validity and the discriminant validity should be assessed. The dominant measure of convergent validity is the average variance extracted (AVE). An AVE value of 0.50 or higher is regard as acceptable [39]. Two criteria have been commonly used for assessing discriminant validity are the Fornell-Larckr criterion and the HTMT criterion [35, 39]. As required by the former criterion, the square root of AVE of each latent variable should be greater than any other correlations in the model [39]. As required by the latter criterion, the HTMT values should be significantly smaller than one [35].

Finally, to determine the multicollinearity in the model, the variance inflation factor (VIF) of the indicators should be assessed. VIF values higher than 5.0 indicate that multicollinearity might play a role [35].

Path coefficients are main outcomes of PLS-SEM which quantify the hypothesized relationships within the structural model [40]. Path coefficients have standardized values from − 1 to + 1, and are interpreted to the same way as standardized regression coefficients. Bootstrapping was performed to evaluate the significance of path coefficients in the model. The R Square value of the general satisfaction was calculated in this study to indicate the percentage of variability accounted for by patients’ experiences of different aspects of care in public hospitals.

The PLS structure equation modeling was carried out in SmartPLS 3.0 [41]. Descriptive analyses were performed in SPSS 22.0 [42]. A 5% significance level was used throughout the analyses.

## Results

### General characteristics

As shown in Table 2, about 40% of outpatients in the survey were men. Persons aged below 44 years accounted for the majority of the sample (65.1%), followed by the middle-aged (45–64 years) and the older (65+ years). In the survey, nearly 30% of respondents had primary-school education or below, over 27% had middle-school education, around 14% had high-school education, and 28% had college-level education or above. About 37% of respondents reported that they had an annual household income below 30,000 Chinese yuan, and 42% had an annual household income at the level of 30,000–80,000 Chinese yuan. Only 1.5% reported that they had an annual household income of more than 300,000 Chinese yuan. Compared to respondents at city-level public hospitals, more respondents at county-level public hospitals were middle-aged, had the middle-school level education or below, and reported that they had the lowest level of annual household income.

**Table 2:** Characteristics of outpatients by city- and county-level hospitals

	Total (*N* = 4782)		City-level hospitals (*N* = 1686)		County-level hospitals (*N* = 3096)	
	*n*	%	*n*	%	*n*	%
Sex						
Men	1957	40.9	704	41.8	1253	40.5
Women	2825	59.1	982	58.2	1843	59.5
Age group						
≤ 44 years	3111	65.1	1143	67.8	1968	63.6
45–64 years	1125	23.5	358	21.2	767	24.8
≥ 65 years	546	11.4	185	11.0	361	11.7
Education level						
Below primary school	771	16.1	209	12.4	562	18.2
Primary school	641	13.4	184	10.9	457	14.8
Middle school	1327	27.7	432	25.6	895	28.9
High school	702	14.7	280	16.6	422	13.6
College and above	1341	28.0	581	34.5	760	24.5
Income group^a^						
First group (lowest)	1809	37.8	516	30.6	1293	41.8
Second group	2043	42.7	749	44.4	1294	41.8
Third group	760	15.9	352	20.9	408	13.2
Fourth group (highest)	74	1.5	35	2.1	39	1.3
Cannot tell	96	2.0	34	2.0	62	2.0

^a^The annual household income of the first income group: < 30,000 Chinese yuan, the second group: 30,000–80,000 Chinese yuan, the third group: 80,000–300,000 Chinese yuan, and the fourth group: ≥ 300,000 Chinese yuan

### General satisfaction by sex, age, socioeconomic status and hospital location

As presented in Table 3, model 1 showed that respondents’ general satisfaction significantly increased with age, the difference between the ≥65 years and the ≤44 years being 0.094. Although there was an education-gradient in general satisfaction according to the coefficients, only the variation between respondents having college-level education and those having an education level below primary school was statistically significant, with a difference of 0.060. In model 2, the variation in general satisfaction by hospital location was analyzed controlling for sex, age, education level, and income group. The age-gradient remained statistically significant, but the variation between educational levels turned statistically insignificant. Compared to those seeking care at city-level hospitals, respondents seeking care at county-level hospitals were significantly less satisfied, with the difference being 0.158. Given such significant difference, the relationship between outpatients’ general satisfaction and their experiences of care was analyzed separately by city- and county-level hospitals.

**Table 3:** Multiple regression analysis on outpatients’ satisfaction with care, on sex, age group, education level, income group and hospital location (*N* = 4728)

	Model 1			Model 2		
	Coefficient	Standard error		Coefficient	Standard error	
Intercept	3.741	0.030	***	3.864	0.034	***
Sex ^a^						
Women	−0.024	0.019		−0.023	0.019	
Age group ^b^						
45–64	0.051	0.024	*	0.053	0.024	*
≥ 65	0.094	0.032	**	0.089	0.031	**
Education level ^c^						
Primary school	0.021	0.036		0.016	0.035	
Middle school	0.037	0.030		0.028	0.030	
High school	0.048	0.034		0.029	0.034	
College and above	0.060	0.030	*	0.038	0.030	
Income group ^d^						
Second group	0.032	0.022		0.022	0.022	
Third group	0.049	0.029		0.026	0.029	
Fourth group	0.026	0.078		0.002	0.078	
Cannot tell	−0.098	0.069		−0.106	0.068	
Hospital location ^e^						
County-level hospitals	–	–		−0.158	0.020	***

*P < 0.05, **P < 0.01, ***P < 0.001

^a^Reference category: men

^b^Rreference category: ≤44 years old

^c^Rreference category: below primary school

^d^Rreference category: first group

^e^Rreference category: city-level hospitals

### Outpatients’ experiences by city- and county-level hospitals

As demonstrated in Table 4, under the aspect of *medical costs*, around 61% of respondents at both city- and county-level hospitals perceived their overall health expenditure and the spending on registration, diagnosis and treatment during this visit as worthwhile or very worthwhile. In the survey, respondents rated their experiences as positive in most aspects of hospital care except *waiting time*, where only 40% respondents at both city- and county-level public hospitals perceived their waiting time for registration and paying fees as relatively short or very short. The differences in the aspects above between city- and county-level hospitals were not statistically significant.

**Table 4:** Outpatients’ experiences of care by city- and county-level hospitals (%)

	City-level hospitals (*N* = 1686)		County-level hospitals (*N* = 3096)		
	Positive responses	Negative responses	Positive responses	Negative responses	
Environment and facilities					
Q25 Environment	85.1	14.9	73.0	27.0	***
Q27 Orders	74.2	25.8	66.8	33.2	***
Q31 Layout	69.7	30.3	62.1	37.9	***
Q36 Facilities	77.8	22.2	71.9	28.1	***
Professional competence					
Q40 Skills	71.5	28.5	53.1	46.9	***
Caring attitudes and emotional support					
Q18 Friendly & respectful	82.3	17.7	78.3	21.7	**
Q19 Patiently	83.2	16.8	80.6	19.4	*
Medical costs					
Q46 Overall expense	60.5	39.5	61.4	38.6	
Q47 Detailed expense	62.6	37.4	61.3	38.7	
Communication and information					
Q13 Explain	69.8	30.2	66.8	33.2	*
Q21 Inform	81.4	18.6	75.1	24.9	***
Q22 Communication	81.9	18.1	78.7	21.3	**
Waiting time					
Q42 Registration & paying fees	41.3	58.7	42.5	57.5	
Q43 Seeing the doctor	33.6	66.4	37.2	62.8	*
General satisfaction					
Q53 General satisfaction	80.8	19.2	70.3	29.7	***

*P < 0.05, **P < 0.01, ***P < 0.001

There were significantly higher percentages of respondents in city-level hospitals who had positive experiences in each aspect of *environment and facilities* than those in county-level hospitals did. Among the four aspects under *environment and facilities*, the difference in respondents’ experiences of hospital environment was the most significant, and 12.1% more respondents in city-level hospitals perceived hospital environment as comfortable and clean than those in county-level hospitals did.

A significantly higher proportion of respondents in city-level hospitals had positive perception of medical staff’s skills than those in county-level hospitals.

In terms of *caring attitudes and emotional support*, respondents in city-level hospitals had significantly higher evaluation than those in county-level hospitals did. Compared with respondents in county-level hospitals, about 4% more respondents in city-level hospitals perceived the attitude of health professionals as friendly and respectful, and 3% more respondents in city-level hospitals felt that doctors listened patiently to their description of conditions during this visit.

Under *communication and information*, similar pattern of variation in outpatient’s experiences between city- and county-level hospitals were found. Among the three aspects under *communication and information*, respondents at both city- and county-level hospitals had the lowest satisfaction with doctors’ explanation of their conditions and relevant issues. Compared with respondents in county-level hospitals, more respondents in city-level hospitals had positive experiences in the provision of information by health professionals on matters needing attention and the communication with doctors during this visit.

In terms of waiting time, there were significantly more respondents in city-level hospitals who felt that the waiting time for seeing the doctor was long or very long compared to those in county-level hospitals.

As to the general satisfaction, there were more respondents in urban areas than those in rural areas who were satisfied with the services provided in public hospitals.

### Model fit assessment

Nearly all model fit indices reached the required thresholds, except that Cronbach’s *α* and *ρ*_*A*_ of *medical costs* were significantly below 0.70. There were two indicators under *medical costs* measured by Q46 and Q47. Since the loading of Q46 was smaller than that of Q47, and Q47 asked for outpatients’ overall perception of expenses, while Q46 inquired about the perception of expenses on specific services such as registration, Q46 was removed. Hence, the final model comprised of indicators regarding the remaining 14 questions. The indices of the final model are presented in Additional file 1: Appendix Table A4-A8. All indices reached their corresponding cut-off values except that Cronbach’s *α* and *ρ*_*A*_ of *environment and facilities* were slightly below 0.70.

### Path models by city- and county-level hospitals

**Fig. 2:**
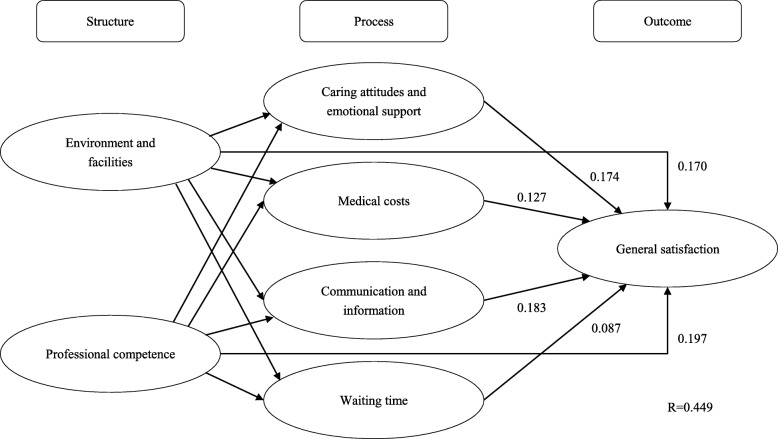
Path coefficients of direct effects in the path model outpatients’ general satisfaction and experiences of care at of city-level public hospitals

As indicated in Fig. 2, among respondents in city-level public hospitals, there were statistically significant direct positive effects from their experiences of *environment and facilities* (path coefficient = 0.170) and from their experiences of *professional competence* (path coefficient = 0.197) to their general satisfaction. Both *environment and facilities* and *professional competence* had significant positive indirect effects on general satisfaction via “process” aspects, with path coefficients being 0.163 and 0.155 respectively. *Communication and information* had the strongest positive direct effect on general satisfaction among the four “process” aspects (path coefficient = 0.183), followed by *caring attitudes and emotional support* (path coefficient = 0.174), *medical costs* (path coefficient = 0.127), and *waiting time* (path coefficient = 0.087). This path model explained 44.9% of variation in the general satisfaction of outpatients at city-level public hospitals.

**Fig. 3:**
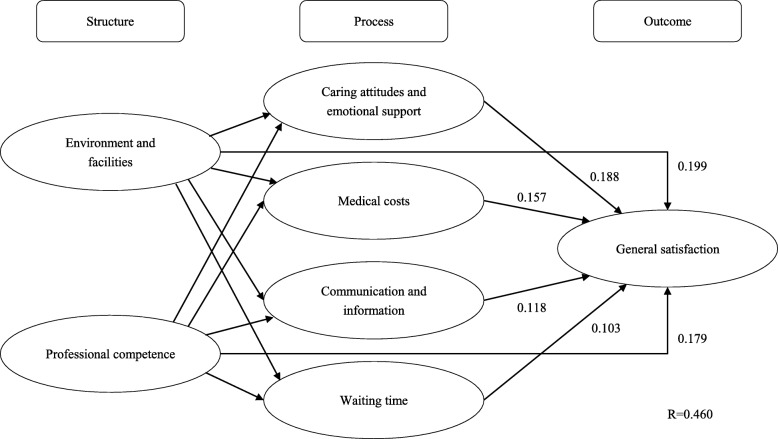
Path coefficients of direct effects in the path model of outpatients’ general satisfaction and experiences of care at county-level public hospitals

As depicted in Fig. 3, among respondents in county-level public hospitals, there were also statistically significant direct positive effects from their experiences of *environment and facilities* (path coefficient = 0.199) and from their experiences of *professional competence* (path coefficient = 0.179) to their general satisfaction. Both *environment and facilities* and *professional competence* had significant positive indirect effects on general satisfaction via “process” aspects, with path coefficients being 0.190 and 0.131 respectively. *Caring attitudes and emotional support* had the strongest positive direct effect on general satisfaction among the four “process” aspects (path coefficient = 0.188), followed by *medical costs* (path coefficient = 0.157), *communication and information* (path coefficient = 0.118), and *waiting time* (path coefficient = 0.103). This path model explained 46.0% of variation in the general satisfaction of outpatients at county-level public hospitals.

## Discussion

This study found that 80.8 and 70.3% of surveyed outpatients were satisfied with health care provided in city- and county-level hospitals respectively, or in other words, in public hospitals in urban and rural areas respectively. As to the specific aspects of outpatients’ experiences of care, outpatients in urban areas reported more positive experiences than those in rural areas in nearly all the aspects of care provided in public hospitals. By applying the PLS method, to the best of our knowledge, this study may be one of the first to provide evidence that 44.9 and 46.0% of outpatients’ general satisfaction with health care in Chinese public hospitals could be explained by outpatients’ experiences of specific aspects of care in the urban and rural area respectively.

The findings of this study demonstrated that although outpatients had substantial negative experiences of *waiting time* at both city-level and county-level public hospitals, the importance of experiences in other aspects of care outweighed the importance of experiences in *waiting time*. To outpatients seeking health care at city-level hospitals, *professional competence* was most important. How they perceive the skills of medical professionals had impacts on not only the general satisfaction with health care provided at hospitals, but also on how they perceive the process of care delivery, including *caring attitudes and emotional support*, *communication and information*, *medical costs*, and *waiting time*. To outpatients seeking health care at county-level hospitals, *environment and facilities* were the most important aspects, influencing both their overall satisfaction and assessment of specific aspects during care delivery.

The difference in the relationship between general satisfaction and experiences of various aspects of care at city- and county-level public hospitals may partly lie in the fact that city-level public hospitals are located in urban areas and all city-level hospitals included in this study are tertiary hospitals. County-level public hospitals, however, are located in rural areas and the majority included in this study are secondary hospitals. Compared with health care facilities in rural areas of China, health care facilities in urban areas are equipped with more and better health resources including financial, human and material resources [18]. This could be one reason why outpatients seeking health services at city-level hospitals in this study reported significantly better experiences in nearly all aspects of care and stressed more on their perception on *professional competence* while less on their experiences of *environment and facilities* than did outpatients at county-level hospitals. Outpatients’ perception of the hospital environment and state of hospital facilities as well as of the level of professional competence of medical staff could be interpreted as important determinants of their general satisfaction with health care in Chinese public hospitals. As these are rated lower among county-level respondents than city respondents, policy makers concerned with the public hospital reform should take this into consideration when allocating health care resources.

To outpatients seeking health services at city-level hospitals, compared with the other aspects relevant to the “process” of care delivery, the aspect of *communication and information* is a more important determinant of respondents’ satisfaction with care. Among the three components under the aspect of *communication and information*, outpatients had the most negative experiences in doctors’ explanation on their illness conditions, followed by the notice of matters needing attention during treatment and the communication with health professionals. These results indicate that at public hospitals in urban areas the factors central to patients’ experiences of *communication and information* are how the medical information is communicated and whether the content is informative. This is in agreement with previous studies, which have demonstrated that patients pay much attention to the provision of information on clinical status and emphasizes the explanation of their clinical status in an easy-to-understand language [43, 44].

To outpatients at county-level hospitals, the aspect of *caring attitudes and emotional support* was a more important determinant of their general satisfaction with care than the other aspects pertaining to the “process” of care delivery. Outpatients have negative perceptions on both the respectfulness and friendliness of health professionals and the patience of doctors. It is indicative from these results that outpatients seeking health care at public hospitals in rural areas may attach importance to a higher level of caring such as being treated with sufficient courtesy and patience from medical staff rather than receiving informative explanation on the condition and treatment. These findings are consistent with previous studies [31, 44].

The above aspects having stronger associations with general satisfaction can be interpreted as opportunities for improvement to further the public hospital reform in China. Although the effect size of *waiting time* on outpatients’ general satisfaction was smaller than those of other aspects of care, it is still of statistical significance. It demonstrates the heavy workload of medical staff at Chinese public hospitals. More effective strategies need to be taken to divert the patient flow to primary health care facilities.

The age of outpatients in this study is generally younger, with over 65% of respondents being below 44 years. Compared with younger patients, older patients usually seek care for chronic diseases and more complex conditions. This finding manifests a prominent issue in China that outpatient visits are concentrated at secondary and tertiary public hospitals regardless of the complexity of the diseases [45]. After large investment in primary health care facilities and implementation of policies on training medical staff, patients still have low trust in the quality of care provided at primary health care facilities [14]. In addition, there is no formal referral system in China. As a result, public hospitals continue to dominate China’s health care delivery [15].

### Methodological considerations

Although PLS has been widely used in research on information systems and strategic management [46, 47], it has been rarely used in the studies on patient satisfaction and experiences. One study reported that patients’ perceived service quality has significant effect on behavioral compliance via patient satisfaction [48]. This study was conducted among 235 inpatients at one Malaysian hospital and since the outcome variable was behavioral compliance, only direct effects of different components of perceived service quality on patient satisfaction were examined. Through using the PLS method, our study contributes to the literature by increasing the knowledge of the relationship between patients’ general satisfaction and patients’ experiences of different aspects of care provided at Chinese public hospitals. The PLS method makes it possible to validate the structural hypotheses on the relationship between the outcome variable and other latent variables and to rank the latent variables according to their impacts on the outcome variable [49]. As in this study the outcome variable – patient satisfaction – is a multidimensional concept, and the relationship between patients’ general satisfaction and their experiences of different aspects of care has not yet been defined, it may be appropriate to use PLS to test the structural hypotheses based on Donabedian’s model and to prioritize the aspects of care provided in Chinese public hospitals by measuring path coefficients.

### Strengths and limitations

To the best of our knowledge, this study may be one of the first to investigate to what extent patient satisfaction with outpatient care in Chinese public hospitals could be explained by their experiences of specific aspects of care. It is based on a large sample, including both urban and rural hospitals and contributes to increasing the understanding of what determines patient satisfaction.

However, there are several limitations to the study. Firstly, a convenience sample was used in the survey and sampling bias should be considered. Participants were more likely to be younger and better educated. Further investigation using population-based samples should be conducted to yield more generalizable findings. Secondly, the five sampled cities in this study were selected based on recommendations from officials in China’s ministry of health. Such selection and recommendation might lead to more favorable perceptions on outpatient services among participants. The research group, therefore, selected another four cities from the same provinces where the public hospital reform had not been initiated before the present study was conducted to acquire a more comprehensive understanding of patient-reported experiences and satisfaction. Besides, data on health care resources were not collected and due to the cross-sectional study design, casual relationships could not be verified in this study. As the results show, outpatients’ general satisfaction at city-level hospitals was significantly higher than that of outpatients at county-level hospitals, even after adjusting for sex, age, education level and income group, which might be caused by the differences in health care resources between urban and rural areas. In future research, data on health care resources such as number of beds, number and educational structure of medical staff, and doctor-nurse ratio should be collected and longitudinal data should be collected to increase the understanding of the differences in outpatients’ general satisfaction with health care in public hospitals between urban and rural areas. The questionnaire designed and used in this study may not be optimal. About 44.9 and 46.0% of the variation in outpatients’ general satisfaction in city-level hospitals and county-level hospitals were explained in this study respectively. Although such results exhibit significant associations between patient satisfaction and patient experiences, it also suggests that there are sources of variation in outpatients’ general satisfaction that have not been included in the model.

## Conclusions

As found in this study, levels of general satisfaction with outpatient care at Chinese public hospitals were higher in urban areas than in rural areas. General satisfaction with outpatient care was explained by partly similar and partly different patient-reported experiences in urban areas and rural areas. Outpatients’ experiences of the professional competence among medical staff were strongly related to their general satisfaction with care in both settings. However, outpatients’ experiences of the hospital environment and the state of hospital facilities were more strongly related to the general satisfaction among respondents in rural than in city areas, whereas their experiences of the communication with and information provided by the medical staff were more strongly related to the general satisfaction among respondents in city than in rural areas. The results of the present study may be useful for policy makers. Further studies, also among inpatients, are needed of patient satisfaction and experiences of care in order to inform and guide the ongoing reform of public hospitals, and to meet the goals of the reform.

## Additional file

Additional file 1: **Appendix Table A1-8.** **1**. Questions and corresponding answering options concerning patients’ experiences of care included in the questionnaire. **2**-**1**. Distribution of outpatients’ experiences of care in Chinese public hospitals under the “structure” category (%) (*N*=4,835). **2**-**2**. Distribution of outpatients’ experiences of care in Chinese public hospital under the “process” category (%) (*N*=4,835). **2**-**3**. Distribution of outpatients’ general evaluation of care in Chinese public hospital (%) (*N*=4835). **3**. The inclusion and exclusion procedures of the 15 questions included in the analyses. **4**. The construct reliability of the final model (*N*=4782). **5**. The convergent validity of the final model (*N*=4782). **6**. The discriminant validity of the final model (the Fornell-Larcker criterion) (*N*=4782). **7**. The discriminant validity of the final model (the HTMT criterion) (*N*=4782). **8**. The multicollinearity statistics of the final model (variance inflation factor, VIF) (*N*=4782). (DOCX 35 kb)

## Abbreviations

AVE: Average Variance Extracted
NHFPC: National Health and Family Planning Commission
PLS: Partial Least Squares
PLS-SEM: Partial Least Squares Structural Equation Modeling
PREMs: Patient-Reported Experience Measures
SEM: Structural Equation Modeling
SRMR: Standardized Root Mean Square Residual
