# Supplementary material for: Exploring pathways to outpatients’ satisfaction with health care in Chinese public hospitals in urban and rural areas using patient-reported experiences
Source: Int J Equity Health. 2019 Feb 6;18:29. doi: 10.1186/s12939-019-0932-3 (PMC6366112; doi:10.1186/s12939-019-0932-3)
Supplement: Supplementary file 1 — Appendix Table A1-8. 1. Questions and corresponding answering options concerning patients’ experiences of care included in the questionnaire. 2-1. Distribution of outpatients’ experiences of care in Chinese public hospitals under the “structure” category (%) (N=4,835). 2-2. Distribution of outpatients’ experiences of care in Chinese public hospital under the “process” category (%) (N=4,835). 2-3. Distribution of outpatients’ general evaluation of care in Chinese public hospital (%) (N=4835). 3. The inclusion and exclusion procedures of the 15 questions included in the analyses. 4. The construct reliability of the final model (N=4782). 5. The convergent validity of the final model (N=4782). 6. The discriminant validity of the final model (the Fornell-Larcker criterion) (N=4782). 7. The discriminant validity of the final model (the HTMT criterion) (N=4782). 8. The multicollinearity statistics of the final model (variance inflation factor, VIF) (N=4782). (DOCX 35 kb) [file 12939_2019_932_MOESM1_ESM.docx]

## Appendix

**Table A1. Questions and corresponding answering options concerning patients’ experiences of care included in the questionnaire**

| **Questions** | **Answering options** |
| --- | --- |
| Q13. Did the doctor explain your condition and related issues concretely? (i.e. state of the illness and the treatment protocol) | 1=Very concretely  2=Concretely  3=Neither concretely nor not concretely  4=Not very concretely  5=Not concretely at all  6=Cannot tell |
| Q14. Do you understand your condition clearly after this visit? | 1=Very clearly  2=Clearly  3=Neither clearly nor not clearly  4=Not very clearly  5=Not clearly at all  6=Cannot tell |
| Q15. Do you understand the treatment protocol of your illness clearly after this visit? | 1=Very clearly  2=Clearly  3=Neither clearly nor not clearly  4=Not very clearly  5=Not clearly at all  6=Cannot tell |
| Q16. Could you get timely responses when you asked questions? | 1=Often  2=Sometimes  3=Never  4=I did not have questions  5=Cannot tell |
| Q17. Could you get timely help when you needed? | 1=Often  2=Sometimes  3=Never  4=I did not need help |
| Q18. Were the medical professionals friendly and respectful during this visit? | 1=Very friendly and respectful  2=Friendly and respectful  3=Moderate  4=Not very friendly or respectful  5= Not friendly or respectful at all |
| Q19. Did the doctor listen to the description of your condition patiently during this visit? | 1=Very patiently  2=Patiently  3=Neither patiently nor not patiently  4=Not very patiently  5=Not patiently at all  6=Cannot tell |
| Q20. Did the doctor ask for your opinions when confirming the treatment protocol? | 1=Yes  2=No  3=I did not need treatment protocols  4=Cannot tell |
| Q21. Did the medical professionals inform you of matters that need attention during the treatment? (e.g. medication plan, rehabilitation, health education) | 1=Very concretely  2=Concretely  3=Neither concretely nor not concretely  4=Not very concretely  5=Not concretely at all |
| Q22. Are you satisfied with the communication between you and the medical professionals during this visit? | 1=Very satisfied  2=Satisfied  3=Neither satisfied nor not satisfied  4=Not very satisfied  5=Not satisfied |
| Q23. Did the medical professionals respect your privacy during the process of diagnosis and treatment? | 1=Yes  2=No  3=I did not care  4=Cannot tell |
| Q24. Did the doctor make sure that there was no other patient in the clinic when you were discussing your condition? | 1=Yes  2=No  3=I did not care  4=Cannot tell |
| Q25. Do you think the hospital environment is comfortable and clean? | 1=Very comfortable and clean  2=Comfortable and clean  3=Moderate  4=Not very comfortable or clean  5=Not comfortable or clean at all |
| Q26. Are the signs clear enough to help you find your destinations in the hospital easily? (e.g. signs for restrooms, departments and floors) | 1=Very clear  2=Clear  3=Neither clear nor not clear  4=Not very clear  5=Not clear at all  6=Cannot tell |
| Q27. Do you think the orders in the hospitals are maintained well? (e.g. the registration desk, the fee payment desk, the pharmacy and the waiting areas in the departments) | 1=Very good  2=Good  3=Moderate  4=Not very good  5=Not good at all  6=Cannot tell |
| Q28. Did the medical professionals check your identity information carefully during the treatment? (e.g. name, bed number) | 1=Always  2=Often  3=Sometimes  4=Never |
| Q29. Did the medical professionals inform you on potential risks and relevant preventative methods during your treatment? (e.g. adverse drug reaction, surgery risks) | 1=Yes  2=No  3=I did not need such information  4=Cannot tell |
| Q30. Does the hospital remind you of personal safety in an appropriate way? (e.g. personal belongings, slippery area, fire, exit passageway, dangerous goods) | 1=Very appropriately  2=Appropriately  3=Neither appropriately nor not appropriately  4=Not very appropriately  5=Not appropriately at all  6=Cannot tell |
| Q31. Is the layout of different departments reasonable so that outpatients can travel less in the hospital? | 1=Very reasonable  2=Reasonable  3=Neither reasonable nor not reasonable  4=Not very reasonable  5=Not reasonable at all  6=Cannot tell |
| Q32. Do you think the medical professionals have done sufficient inquiry and medical check-up? | 1=Very sufficient  2=Sufficient  3=Neither sufficient nor not sufficient  4=Not very sufficient  5=Not sufficient at all  6=Cannot tell |
| Q33. Did the medical professionals tell you that you need to revisit or that they would follow you up? | 1=Yes  2=No  3=Cannot tell |
| Q34. Do you think the medical professionals followed the standard procedures during this visit? | 1=Very standard  2=Standard  3=Neither standard nor not standard  4=Not very standard  5=Not standard at all  6=Cannot tell |
| Q35. Do you know that there are other ways of registering such as online and telephone registering besides the registration desk? | 1=Yes  2=No  3=I did not care |
| Q36. Could the facilities in the hospital satisfy your need? (e.g. seats in the resting areas, supply of hot water, wheelchairs, entertainment equipment, parking lots) | 1=Very satisfied  2=Satisfied  3=Neither satisfied nor not satisfied  4=Not very satisfied  5=Not satisfied at all  6=Cannot tell |
| Q37. Do you know how to make complaints and suggestions to the hospital? | 1=Yes  2=No  3=I did not care |
| Q38. (Only ask inpatients) Could the treatment relief your symptoms and pain effectively? | 1=Very effectively  2=Effectively  3=Neither effectively nor not effectively  4=Not very effectively  5=Not effectively at all  6=Cannot tell |
| Q39. (Only ask inpatients) Compare to your expectation, how do you evaluate the treatment outcome so far? | 1=Very good  2=Good  3=Moderate  4=Not very good  5=Not good at all  6=Cannot tell |
| Q40. What do you think of the skills of the medical professionals in this hospital? | 1=Very high  2=High  3=Moderate  4=Not very high  5=Not high at all  6=Cannot tell |
| Q41. Do you think that the medical equipment in this hospital can satisfy your need of diagnosis and treatment? (e.g. CT, MRI) | 1=Very satisfied  2=Satisfied  3=Neither satisfied nor not satisfied  4=Not very satisfied  5=Not satisfied at all  6=Cannot tell |
| Q42. Do you think the waiting time for registration and paying fees was reasonable? | 1=Very short  2=Relatively short  3=Neither short nor long  4=A little bit long  5=Very long  6=Cannot tell |
| Q43. Do you think the waiting time for seeing the doctor was reasonable? | 1=Very short  2=Relatively short  3=Neither short nor long  4=A little bit long  5=Very long  6=Cannot tell |
| Q44. Do you think the waiting time for examination was reasonable? | 1=Very short  2=Relatively short  3=Neither short nor long  4=A little bit long  5=Very long  6=No need for examination |
| Q45. Do you think the waiting time for getting medicines was reasonable? | 1=Very short  2=Relatively short  3=Neither short nor long  4=A little bit long  5=Very long  6=No need for getting medicines  7=Cannot tell |
| Q46. Do you think the amount of money you spent this time was worthwhile regarding to your condition? | 1=Very worthwhile  2=Worthwhile  3=Neither worthwhile nor not worthwhile  4=Not very worthwhile  5=Not worthwhile at all  6=Cannot tell |
| Q47. Do you think the amount of money you spent on registration, diagnosis and treatment this time was reasonable? | 1=Very reasonable  2=Reasonable  3=Neither reasonable nor not reasonable  4=Not very reasonable  5=Not reasonable at all  6=Cannot tell |
| Q48. Do you think the amount of money you spent on medicines this time was reasonable? | 1=Very reasonable  2=Reasonable  3=Neither reasonable nor not reasonable  4=Not very reasonable  5=Not reasonable at all  6=Did not buy medicines  7=Cannot tell |
| Q49. Do you think the amount of money you spent on tests and examinations this time was reasonable? | 1=Very reasonable  2=Reasonable  3=Neither reasonable nor not reasonable  4=Not very reasonable  5=Not reasonable at all  6=Did not take tests or examinations  7=Cannot tell |
| Q50. Do you think the tests and examinations you took this time were necessary? | 1=Yes  2=No  3=Hard to explain or did not take testes or examinations  4=Cannot tell |
| Q51. Do you think the medicines the doctor prescribed this time were reasonable? | 1=Very reasonable  2=Reasonable  3=Neither reasonable nor not reasonable  4=Not very reasonable  5=Not reasonable at all  6=Hard to explain or did not have prescribed medicines |
| Q52. What do you think of the amount of money you spent this time that cannot be reimbursed? | 1=Very small  2=Relatively small  3=Neither small nor large  4=large  5=Very large  6=I do not have health insurance or it is hard to explain  7=Cannot tell |
| Q53. In general are you satisfied with the services in this hospital? | 1=Very satisfied  2=Satisfied  3=Neither satisfied nor not satisfied  4=Not satisfied  5=Not satisfied at all |
| Q54. Would you still go to this hospital if you need to see the doctor next time? | 1=Yes  2=No  3=Not sure |
| Q55. Would you recommend this hospital to your relatives and friends if they are sick? | 1=Yes  2=No  3=Not sure |

**Table A2-1. Distribution of outpatients’ experiences of care in Chinese public hospitals under the “structure” category (%) (N=4,835)**

| ***Environment and facilities*** | | | | | |
| --- | --- | --- | --- | --- | --- |
| **Most positive** | **Positive** | **Medium** | **Negative** | **Most negative** | **Cannot tell** |
| Q25. Do you think the hospital environment is comfortable and clean? | | | | | |
| 13.7 | 63.6 | 20.7 | 1.5 | 0.6 | 0.0 |
| Q26. Are the signs clear enough to help you find your destinations in the hospital easily? | | | | | |
| 22.8 | 63.5 | 9.6 | 3.7 | 0.5 | 0.0 |
| Q27. Do you think the orders in the hospitals are maintained well? | | | | | |
| 14.3 | 55.2 | 21.5 | 7.4 | 1.6 | 0.0 |
| Q30. Does the hospital remind you of personal safety in an appropriate way? | | | | | |
| 14.7 | 50.3 | 19.9 | 12.5 | 2.5 | 0.0 |
| Q31. Is the layout of different departments reasonable so that outpatients can travel less in the hospital? | | | | | |
| 7.3 | 57.5 | 23.9 | 9.4 | 1.9 | 0.0 |
| Q36. Could the facilities in the hospital satisfy your need? | | | | | |
| 11.7 | 62.1 | 19.9 | 5.4 | 0.8 | 0.0 |
| Q41. Do you think that the medical equipment in this hospital can satisfy your need of diagnosis and treatment? | | | | | |
| 9.5 | 62.3 | 16.5 | 2.5 | 0.4 | 8.8 |
| **Yes** | **No** | **I did not care** | | | |
| Q35. Do you know that there are other ways of registering such as online and telephone registering besides the registration desk? | | | | | |
| 43.4 | 40.6 | 16.1 | | | |
| Q37. Do you know how to make complaints and suggestions to the hospital? | | | | | |
| 36.7 | 46.1 | 17.2 | | | |
| ***Professional competence*** | | | | | |
| **Most positive** | **Positive** | **Medium** | **Negative** | **Most negative** | **Cannot tell** |
| Q32. Do you think the medical professionals have done sufficient inquiry and medical check-up? | | | | | |
| 11.3 | 63.7 | 21.2 | 3.4 | 0.5 | 0.0 |
| Q34. Do you think the medical professionals followed the standard procedures during this visit? | | | | | |
| 13.5 | 70.0 | 14.8 | 1.2 | 0.3 | 0.1 |
| Q40. What do you think of the skills of the medical professionals in this hospital? | | | | | |
| 6.5 | 52.8 | 37.0 | 2.4 | 0.8 | 0.5 |
| ***Moral of medical staff*** | | | | | |
| **Yes** | **No** | **Cannot tell or not applicable** | | | |
| Q50. Do you think the tests and examinations you took this time were necessary? | | | | | |
| 61.1 | 5.2 | 33.8 | | | |
| **Most positive** | **Positive** | **Medium** | **Negative** | **Most negative** | **Cannot tell or not applicable** |
| Q51. Do you think the medicines the doctor prescribed this time were reasonable? | | | | | |
| 8.6 | 62.5 | 12.7 | 2.8 | 0.7 | 12.7 |

**Table A2-2. Distribution of outpatients’ experiences of care in Chinese public hospital under the “process” category (%) (N=4,835)**

| ***Caring attitudes and emotional support*** | | | | | | |
| --- | --- | --- | --- | --- | --- | --- |
| **Most positive** | **Positive** | **Medium** | **Negative** | **Most negative** | **Cannot tell** | |
| Q18. Were the medical professionals friendly and respectful during this visit? | | | | | | |
| 22.3 | 57.5 | 17.7 | 2.0 | 0.5 | 0.0 | |
| Q19. Did the doctor listen to the description of your condition patiently during this visit? | | | | | | |
| 20.3 | 61.2 | 15.4 | 2.6 | 0.5 | 0.0 | |
| **Yes** | **No** | **I did not care** | | **Cannot tell** | | |
| Q23. Did the medical professionals respect your privacy during the process of diagnosis and treatment? | | | | | | |
| 75.0 | 6.7 | 18.2 | | 0.1 | | |
| Q24. Did the doctor make sure that there was no other patient in the clinic when you were discussing your condition? | | | | | | |
| 45.3 | 35.5 | 19.2 | | 0.0 | | |
| ***Medical costs*** | | | | | | |
| **Most positive** | **Positive** | **Medium** | **Negative** | **Most negative** | **Not applicable** | **Cannot tell** |
| Q46. Do you think the amount of money you spent this time was worthwhile regarding to your condition? | | | | | | |
| 5.1 | 55.9 | 31.0 | 6.7 | 1.0 | 0.0 | 0.3 |
| Q47. Do you think the amount of money you spent on registration, diagnosis and treatment this time was reasonable? | | | | | | |
| 5.6 | 55.9 | 27.1 | 9.7 | 1.3 | 0.0 | 0.3 |
| Q48. Do you think the amount of money you spent on medicines this time was reasonable? | | | | | | |
| 4.8 | 46.3 | 28.0 | 12.7 | 2.1 | 5.8 | 0.3 |
| Q49. Do you think the amount of money you spent on tests and examinations this time was reasonable? | | | | | | |
| 4.3 | 39.8 | 25.0 | 10.6 | 2.0 | 18.0 | 0.3 |
| **Most positive** | **Positive** | **Medium** | **Negative** | **Most negative** | **Do not have health insurance or cannot tell** | |
| Q52. What do you think of the amount of money you spent this time that cannot be reimbursed? | | | | | | |
| 4.4 | 8.6 | 19.2 | 6.0 | 1.1 | 60.7 | |

**Table A2-2. Distribution of outpatients’ experiences of care in Chinese public hospital under the “process” category (%) (N=4,835) (Continued)**

| ***Communication and information*** | | | | | | | | | |
| --- | --- | --- | --- | --- | --- | --- | --- | --- | --- |
| **Most positive** | **Positive** | **Medium** | | **Negative** | | **Most negative** | **Cannot tell** | | |
| Q13. Did the doctor explain your condition and related issues concretely? | | | | | | | | | |
| 13.7 | 54.1 | 23.1 | | 7.7 | | 1.3 | 0.0 | | |
| Q14. Do you understand your condition clearly after this visit? | | | | | | | | | |
| 11.2 | 60.6 | 18.1 | | 9.5 | | 0.6 | 0.0 | | |
| Q15. Do you understand the treatment protocol of your illness clearly after this visit? | | | | | | | | | |
| 11.5 | 61.2 | 17.2 | | 9.2 | | 0.8 | 0.0 | | |
| Q21. Did the medical professionals inform you of matters that need attention during the treatment? | | | | | | | | | |
| 19.8 | 57.6 | 14.9 | | 6.1 | | 1.7 | 0.0 | | |
| Q22. Are you satisfied with the communication between you and the medical professionals during this visit? | | | | | | | | | |
| 16.2 | 63.6 | 17.1 | | 2.2 | | 0.9 | 0.0 | | |
| **Often** | **Sometimes** | **Never** | | **Not applicable** | | **Cannot tell** | | | |
| Q16. Could you get timely responses when you asked questions? | | | | | | | | | |
| 66.8 | 27.1 | 1.4 | | 4.7 | | 0.0 | | | |
| Q17. Could you get timely help when you needed? | | | | | | | | | |
| 64.6 | 27.0 | 1.7 | | 6.6 | | 0.0 | | | |
| **Always** | **Often** | **Sometimes** | | **Never** | | | | | |
| Q28. Did the medical professionals check your identity information carefully during the treatment? | | | | | | | | | |
| 72.5 | 19.8 | 6.7 | | 1.0 | | | | | |
| **Yes** | **No** | **Not applicable** | | | | **Cannot tell** | | | |
| Q20. Did the doctor ask for your opinions when confirming the treatment protocol? | | | | | | | | | |
| 79.3 | 12.2 | 8.5 | | | | 0.0 | | | |
| Q29. Did the medical professionals inform you on potential risks and relevant preventative methods during your treatment? | | | | | | | | | |
| 77.5 | 14.9 | 7.6 | | | | 0.0 | | | |
| Q33. Did the medical professionals tell you that you need to revisit or that they would follow you up? | | | | | | | | | |
| 79.6 | 20.2 | 0.0 | | | | 0.1 | | | |
| ***Efficiency and coordination of care*** | | | | | | | | | |
| **Most positive** | **Positive** | **Medium** | **Negative** | | **Most negative** | | | **Not applicable** | **Cannot tell** |
| Q42. Do you think the waiting time for registration and paying fees was reasonable? | | | | | | | | | |
| 6.9 | 35.2 | 34.8 | 18.0 | | 5.0 | | | 0.0 | 0.0 |
| Q43. Do you think the waiting time for seeing the doctor was reasonable? | | | | | | | | | |
| 5.7 | 30.3 | 40.0 | 18.7 | | 5.3 | | | 0.0 | 0.0 |
| Q44. Do you think the waiting time for examination was reasonable? | | | | | | | | | |
| 4.2 | 20.7 | 33.2 | 18.0 | | 6.3 | | | 17.5 | 0.0 |
| Q45. Do you think the waiting time for getting medicines was reasonable? | | | | | | | | | |
| 10.9 | 41.2 | 32.6 | 7.2 | | 1.7 | | | 6.3 | 0.0 |

**Table A2-3. Distribution of outpatients’ general evaluation of care in Chinese public hospital (%) (N=4,835)**

| **Most positive** | **Positive** | **Medium** | **Negative** | **Most negative** |
| --- | --- | --- | --- | --- |
| Q53. In general are you satisfied with the services in this hospital? | | | | |
| 9.8 | 64.3 | 23.2 | 2.2 | 0.5 |
| **Yes** | **No** | **Not sure** | | |
| Q54. Would you still go to this hospital if you need to see the doctor next time? | | | | |
| 77.8 | 2.8 | 19.4 | | |
| Q55. Would you recommend this hospital to your relatives and friends if they are sick? | | | | |
| 67.5 | 5.5 | 27.0 | | |

**Table A3. The inclusion and exclusion procedures of the 15 questions included in the analyses.**

| **Aspects** | **Questions inclusion and exclusion procedures** |
| --- | --- |
| *Environment and facilities* | Under this aspect, the care measured by questions covering Q26, Q30, Q35 and Q37 in the original questionnaire were not mentioned by patients in the previous study. As shown in Appendix 2, nearly 9% outpatients chose the “cannot tell” answering option of Q41 in the survey, which exhibited relatively low validity of this item. These five questions, therefore, were excluded from analysis. |
| *Professional competence* | In literature questions regarding concrete procedures performed by medical staff were used to measure professional competence. However, according to findings in the previous study of the research group, patients in Chinese public hospitals were mostly concerned with the capacity, reputation, experience and professional title of medical staff. These were more general concepts. As a result, Q32 and Q34 pertaining to more detailed procedures were excluded. Only Q40 under this aspect was included in the analysis. |
| *Moral of medical staff* | Only two questions were related to this aspect. However, about 13% and 34% outpatients in the survey chose the answering option of “cannot tell or not applicable”, respectively. The research group could not separate those who found it difficult to choose an answer from those who did not receive relevant services. Consequently, both questions were excluded from analysis. |
| *Caring attitudes and emotional support* | Under this aspect, privacy protection was mentioned only once in the previous study. The format of answering options of the two questions concerning privacy protection, i.e. Q23 and Q24, was different from the others in the questionnaire. Over 18% outpatients chose the “I did not care” option. Thus, these two questions were excluded from analysis. |
| *Medical costs* | About 6% and 18% outpatients chose the “not applicable” answering option of Q48 and Q49, respectively. For Q52, it contained one answering option that included both “without health insurance” and “cannot tell”, and over 60% outpatients chose this answer. These three questions, therefore, were not included in the study. |
| *Communication and information* | Under this aspect, Q17, Q28, Q29 and Q33 were not mentioned by patients in the previous study. Both Q14 and Q15 described patients’ own understanding of their diseases and the treat protocols, which were not perceptions of services provided by medical staff. For Q16 and Q20, about 5% and 9% outpatient chose the “not applicable” answering option in the survey, respectively. As a result, these eight questions were excluded from analysis. |
| *Waiting time* | The initial title of this aspect was efficiency and coordination of care, but all questions used in the questionnaire were pertaining to waiting time. The title of this aspect was changed into waiting time accordingly. About 18% and 6% outpatients chose the “not applicable” answering option of Q44 and Q45, respectively. Thus, these two questions were not included in the analysis. |
| *General satisfaction* | In the questionnaire, Q53 asking for patient’s general satisfaction with the services provided in the hospital. Therefore, this question was included in the analysis and served as the measure of patients’ general satisfaction. |

**Table A4. The construct reliability of the final model (N=4782)**

| **Latent variable** | ***ρ_A_*** | **Cronbach’s *α*** | **Composite Reliability** |
| --- | --- | --- | --- |
| Environment and facilities | 0.685 | 0.687 | 0.809 |
| Professional competence | 1.000 | 1.000 | 1.000 |
| Caring attitudes and emotional support | 0.794 | 0.798 | 0.907 |
| Medical costs | 1.000 | 1.000 | 1.000 |
| Communication and information | 0.751 | 0.777 | 0.856 |
| Waiting time | 0.748 | 0.748 | 0.888 |
| General satisfaction | 1.000 | 1.000 | 1.000 |

**Note: The threshold values of *ρ_A_*, Cronbach’s *α* and composite reliability are 0.70. All values are above the thresholds except for those of environment and facilities.**

**Table A5. The convergent validity of the final model (N=4782)**

| **Latent variable** | **Average Variance Extracted (AVE)** |
| --- | --- |
| Environment and facilities | 0.515 |
| Professional competence | 1.000 |
| Caring attitudes and emotional support | 0.829 |
| Medical costs | 1.000 |
| Communication and information | 0.666 |
| Waiting time | 0.799 |
| General satisfaction | 1.000 |

**Note: Since the AVE values are all above the 0.50 criterion, it demonstrates adequate convergent validity of the final model.**

**Table A6. The discriminant validity of the final model (the Fornell-Larcker criterion) (N=4782)**

| **Latent variable** | **Environment and facilities** | **Professional competence** | **Caring attitudes and emotional support** | **Medical costs** | **Communication and information** | **Waiting time** | **General satisfaction** |
| --- | --- | --- | --- | --- | --- | --- | --- |
| **Environment and facilities** | 0.717 |  |  |  |  |  |  |
| **Professional competence** | 0.474 | 1.000 |  |  |  |  |  |
| **Caring attitudes and emotional support** | 0.471 | 0.428 | 0.911 |  |  |  |  |
| **Medical costs** | 0.364 | 0.374 | 0.357 | 1.000 |  |  |  |
| **Communication and information** | 0.473 | 0.442 | 0.684 | 0.372 | 0.816 |  |  |
| **Waiting time** | 0.386 | 0.265 | 0.259 | 0.256 | 0.223 | 0.894 |  |
| **General satisfaction** | 0.527 | 0.504 | 0.528 | 0.426 | 0.516 | 0.334 | 1.000 |

**Note: Each underlined value is the square root of the average variance extracted (AVE) of each latent variable, i.e. the diagonal value. Since the diagonal values are greater than any other correlations, it demonstrates adequate discriminant validity of the final model.**

**Table A7. The discriminant validity of the final model (the HTMT criterion) (N=4782)**

| **Latent variable** | **Environment and facilities** | **Professional competence** | **Caring attitudes and emotional support** | **Medical costs** | **Communication and information** | **Waiting time** |
| --- | --- | --- | --- | --- | --- | --- |
| **Professional competence** | 0.572 |  |  |  |  |  |
| **Caring attitudes and emotional support** | 0.635 | 0.480 |  |  |  |  |
| **Medical costs** | 0.439 | 0.374 | 0.400 |  |  |  |
| **Communication and information** | 0.648 | 0.505 | 0.869 | 0.425 |  |  |
| **Waiting time** | 0.540 | 0.306 | 0.334 | 0.296 | 0.291 |  |
| **General satisfaction** | 0.636 | 0.504 | 0.591 | 0.426 | 0.584 | 0.386 |

**Note: Since the HTMT values are all smaller than one, it demonstrates adequate discriminant validity of the final model.**

**Table A8. The multicollinearity statistics of the final model (variance inflation factor, VIF) (N=4782)**

| **Latent variable** | **Caring attitudes and emotional support** | **Medical costs** | **Communication and information** | **Waiting time** | **General satisfaction** |
| --- | --- | --- | --- | --- | --- |
| **Environment and facilities** | 1.289 | 1.289 | 1.289 | 1.289 | 1.636 |
| **Professional competence** | 1.289 | 1.289 | 1.289 | 1.289 | 1.475 |
| **Caring attitudes and emotional support** |  |  |  |  | 2.035 |
| **Medical costs** |  |  |  |  | 1.295 |
| **Communication and information** |  |  |  |  | 2.068 |
| **Waiting time** |  |  |  |  | 1.208 |

**Note: Since the VIF values are all under the 5.0 criterion, it demonstrates that multicollinearity is not present in the final model.**
